# The Role and Significance of Trace Elements in Oral Submucosal Fibrosis

**DOI:** 10.7759/cureus.62688

**Published:** 2024-06-19

**Authors:** Kaushiki Saoji, Amit Reche

**Affiliations:** 1 Department of Public Health and Dentistry, Datta Meghe Institute of Higher Education and Research, Wardha, IND

**Keywords:** anaemia, chewing nuts, trace elements, oral submucous fibrosis (osmf), oral cancer

## Abstract

Oral cancer represents the greatest cause of cancer-related morbidity and death in the majority of areas where tobacco use is common. There is accumulating evidence that the quantities of essential elements change with the beginning and progression of malignant disease. Essential elements operate as a micro-source in numerous metabolic reactions. To provide an area for the particularly important or necessary trace elements like selenium, excess of iodine (I), iron (Fe), zinc (Zn), and other minor elements other trace element disorders such as oculopharyngeal muscular dystrophy (OPMD) are treated using antioxidants. However, even elevated ingestion of these trace elements such as copper could lead to oral submucosa disorder and the advancement of diversified oral diseases and conditions. Trace element enzymes play a very vital role in a variety of biological and chemical events. In redox operations, some trace elements are complicated. Oral potentially malignant fibrosis has a profound influence on the body and early oral symptoms are frequently used to diagnose such disorders. The objective is to elaborate on the role and significance of various trace elements in oral submucous fibrosis.

## Introduction and background

Micronutrients play a therapeutic role in coping with chronic oxidative stress in biological tissues, as well as supporting regeneration processes and fighting infections. The elements that contain the creation of the covalent bonds, which happen to be an essential component of tissues and semi-major elements, are known as abundant elements [1]. They comprise trace elements, vitamins, and antioxidants. The concept of *trace elements* refers to substances with excessive bioavailability that are present in nature and the immediate surroundings in extremely small amounts and hurt living organisms [2]. Zinc (Zn), copper (Cu), selenium (Se), chromium (Cr), cobalt (Co), iodine (I), manganese (Mn), and molybdenum (Mo) are all trace elements that are essential and vital. The lack of a trace element is associated with a group of symptoms, given that every trace element is connected to a variety of enzymes, rather than just one clinical sign [3]. A chronic, precancerous condition known as oral submucous fibrosis (OSMF) has been shown to primarily affect people in South and Southeast Asia, particularly those from the Indian subcontinent [4]. With an estimated 2.5 million people infected, it has now turned into an epidemic in India [5-6]. Among persons of Asian descent in particular, OSMF has become one of the most common potentially malignant disorders (PMDs) [7].

The pathophysiology of this syndrome has been linked to areca nut chewing, chili use, hereditary predisposition, nutritional deficiencies, autoimmune, and abnormalities of collagen. Arecoline, a component of areca nuts, is the most frequently cited etiology for OSMF [4]. Early on in OSMF, the oral mucosa becomes pale and slightly opaque. Mucosal fibrosis, most commonly in the palate, buccal mucosa, and facial pillars, causes rigidity in late cases of OSMF. Among other symptoms, progressive fibrosis can lead to difficulties blowing out candles or whistling, as well as difficulties swallowing.

Histopathologically, the early stages of OSMF are characterized by polymorphonuclear leukocyte infiltration, epithelial hyperplasia, thicker collagen bundles, and a low number of primary fibroblasts. Epithelial atrophy, dense collagen bundles and sheets, reduced vascularity, absence of edema, and a decrease in inflammatory cells (plasma and lymphocytes) are characteristics of advanced cases [8]. Thick bands of sub-epithelial hyalinization that permeate the submucosal tissues and replace fibrovascular or fat tissue are also indicative of advanced cases [6]. OSMF is a known, potentially cancerous condition. Malignant transformation rates as high as 7.6% have been reported from the Indian subcontinent over 17 years [6].

The gold standard for determining an OSMF diagnosis and prognosis is a biopsy. It takes a long time, is invasive, and some patients experience psychological distress as a result. Therefore, the test must be rapid, easy to interpret, minimally invasive, simple to do, and cost-effective while still being highly conclusive in its diagnosis. Other methods of diagnosis have been used as a result, including cytochemical indicators, hematological profiles, analysis of trace elements, circulating immune complexes, and cytogenetic studies [9]. Copper and iron are two of the countless trace metals necessary for the operation of several enzymes.

An essential trace element in the human body is copper. It's found in a lot of important enzymes, like cytochrome, metallothionein, calcium oxidase, superoxide dismutase, and lysyl oxidase. It has been established that iron, which is present in many hemoproteins such as cytochrome, myoglobin, and hemoglobin, is necessary for the maturation of epithelium [9]. Consequently, biochemical variations in the serum concentrations of iron and copper in premalignant patients can help determine the optimal treatment plan, the stage of the disease, and their prognosis.

## Review

Objectives

Trace elements are minerals that the body requires in minute amounts but are necessary for a variety of physiological activities. Trace element imbalances or deficiencies can have adverse impacts on health and may be associated with the development of OSMF. To better understand their vital roles and determine their significance in the onset, severity, and management of this possibly premalignant oral condition, investigators are examining trace elements in the context of OSMF. Se, Zn, Cu, and other trace elements are crucial for collagen synthesis, immunological response, tissue repair, and antioxidant defense. Their importance stems from the possibility that they could act as diagnostic and prognostic markers, direct treatment approaches, and provide information about preventive measures. Researchers hope to improve OSMF diagnosis, treatment, and prevention methods by thoroughly comprehending the effect of trace elements on this disorder.

Role of trace elements

**Figure 1:**
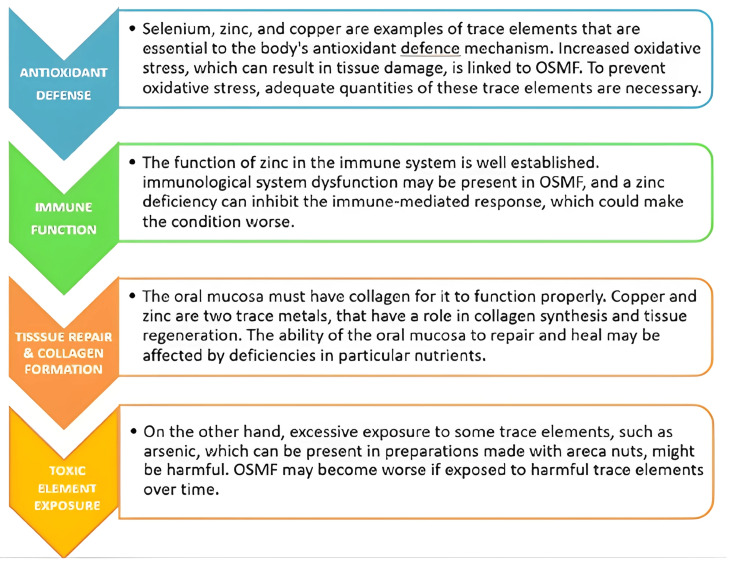
The role of trace elements. Image credit: All authors. OSMF, oral submucous fibrosis

It's necessary to remember that a study regarding the function of trace elements in OSMF is currently taking place, and the exact processes by which these elements regulate the illness are not yet fully understood. Nutritional deficiencies may exacerbate the condition and prevent the body from repairing and regeneration the OSMF that affects the oral tissues. Multiple deficiencies can influence the levels of various trace elements. The management and prevention of OSMF depend mainly on a balanced diet rich in these trace elements, appropriate medical care, and avoiding risk factors, including areca nut and tobacco use. Consult a healthcare provider for an appropriate diagnosis and treatment plan if you believe you have OSMF or are at risk. The role of trace elements is antioxidant defense, immune function, tissue repair, collagen formation, and toxic element exposure (Figure 1).

Significance of trace elements

The importance of trace elements in OSMF is because of their ability to affect the onset, severity, and course of OSMF. It can be used as a diagnostic and prognostic marker. Trace element assessments can be used as diagnostic and prognosis indicators in people with OSMF. The presence or advancement of the illness may be indicated by deficiencies or imbalances in certain components.

It can be used as a therapeutic strategy. Treatment strategies can be guided by an understanding of the function of trace elements. For instance, adding antioxidant supplements like Se or Zn should be considered for reducing oxidative stress. On the other hand, identifying exposure to harmful trace elements may cause actions that reduce or prevent it. It also helps as a preventive measure. Recognizing the association between the use of areca nut preparations and exposure to harmful trace elements can help public health initiatives discourage their use, potentially reducing the risk of OSMF in vulnerable groups.

Classification

World Health Organization Classification

Nineteen trace elements were divided into three classes in 1973: (1) Cu, Zn, Se, Cr, Co, I, Mn, and Mo are essential elements; (2) likely essential elements; and (3) potentially hazardous substances [10].

The Classification of Frieden

Frieden created a biological classification of these elements in 1981 [11] based on the amount of trace elements found in tissues: (1) Essential trace elements include boron, Co, Cu, I, Fe, Mn, Mo, and Zn. (2) It's likely that the trace elements vanadium, nickel, Se, fluoride, and Cr are necessary. (3) Bromine, lithium, silicon, tin, and titanium are examples of physical enhancers.

Importance of trace elements

Trace elements play an important role in body mechanisms as well as in day-to-day activities. It plays an important role in collagen metabolism. It also helps in the antioxidant defense mechanism. It helps in maintaining the immunity of the body. During any injury to the body, it helps in wound healing. It also helps in DNA repair.

Role in Collagen Metabolism

For the metabolism of collagen, an important constituent of connective tissues, trace metals like Zn and Cu are required. The imbalances in these trace elements can affect collagen synthesis and breakdown.

Antioxidant defense: Se and Mn are two trace minerals that are essential for antioxidant defense processes. Due to betel nut and cigarette use, OSMF is linked to increased oxidative stress, which can cause cell damage. These trace elements should be present in adequate amounts to help prevent oxidative damage.

Immune function: A healthy immune system depends on trace elements like Fe, Zn, and Cu. Given that OSMF may impair immunity.

Wound healing: Zn and other trace metals are required for wound healing. The management of OSMF requires effective wound healing because the development of ulcers and lesions in the oral mucosa is prevalent.

DNA repair: One trace element involved in DNA repair mechanisms is Se. It is thought that betel nut products and smoking cause DNA damage and mutations that aid in OSMF development. Sufficient levels of Se may lower risk and help repair DNA.

Mineral deficiencies: Nutritional deficiencies are frequently caused by OSMF, and these deficiencies can have an impact on dental health as well as general health. A sufficient trace element intake can lessen the effects of these shortages. Trace element enzymes play a significant role in a diversified of biological and chemical events [12]. Trace element enzymes are involved in a wide range of biological and chemical activities. For instance, when food products are burned. Some biological compounds have structural roles that have an impact on their stability and three-dimensional structures.

Various trace elements

**Figure 2:**
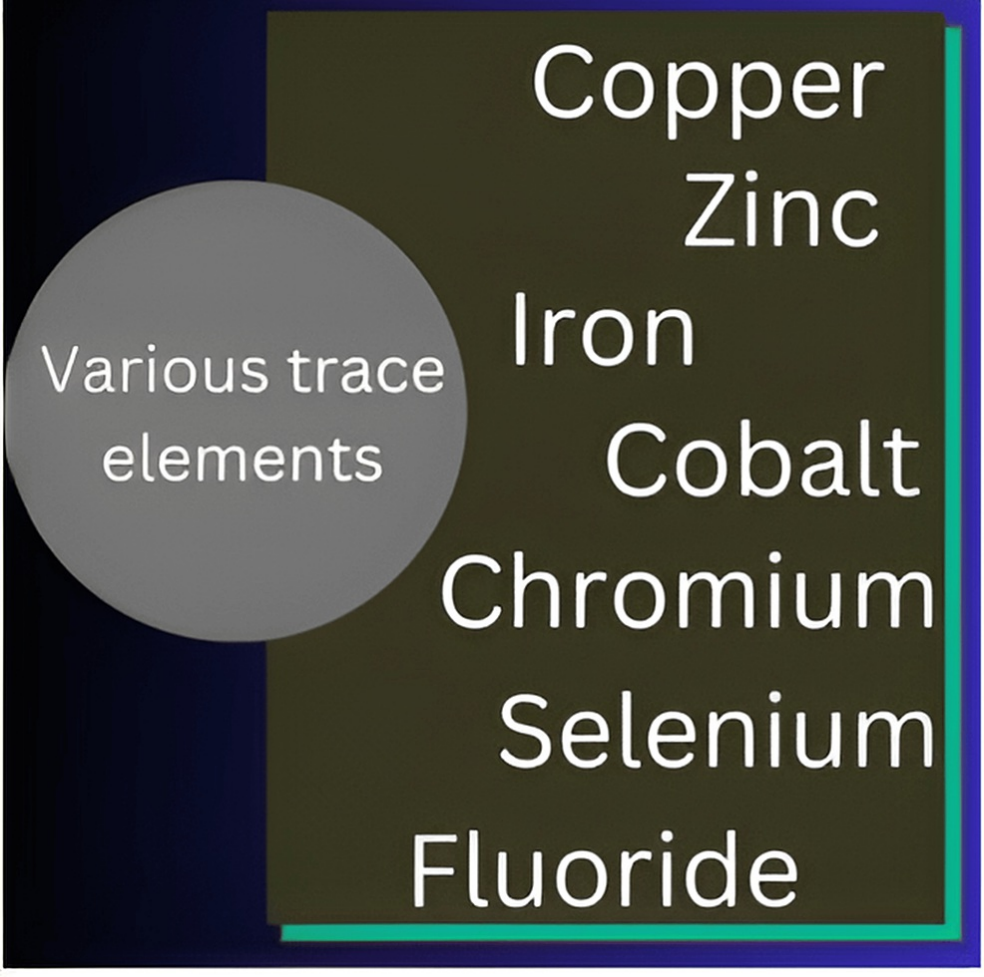
Various trace elements. Image credit: All authors.

The various trace elements are presented in Figure 2.

Copper

One important component of connective tissue, collagen, is made possible by the trace element Cu. Abnormal collagen deposition is a hallmark of OSMF, and Cu may be involved in this process. Cu is the third most common trace metal in humans, with 75-100 mg found in the body [13]. Cu is stored in plasma and erythrocytes, which make up the bulk of human blood [14]. Ceruloplasmin, which is transported into the plasma, is activated in the bile and its metabolism is regulated there [15]. Ninety percent of the Cu in blood is regulated by ceruloplasmin [16].

Biological processes: Some metabolic enzymes cannot function properly without Cu. Numerous antioxidants and cellular energy are related to Cu. Through neurotransmitters, Cu controls the production and metabolism of hormones such as melatonin. Cu concentrations also influence the production of collagen, the development of red blood cells, and the oxidation of fat. Additionally, Fe absorption and vitamin C function depend on Cu [17,18,19,20].

Zinc

Zn is an additional trace element that aids in wound healing and tissue repair. Its absence may impair tissue regeneration and the immune system, which could contribute to the initiation and progression of OSMF. The human body contains two to four grams of Zn [21]. Zn is stored in the liver, muscles, brain, eyes, prostate, and eyes [22]. Except for Fe, which is the second most common metal in living things, it is the only metal present in every family of enzymes [23]. *Transferrin* (10%) and *albumin *(60%) are linked and carry Zn in blood plasma [24].

Biological processes: Zn has many applications in life, but it can be divided into three categories: structural, regulatory, and catalytic [25]. It affects cell division, protein synthesis, wound healing, and immune function. Zn can be utilized for maintaining the sensations of taste and smell in good condition. It facilitates balanced development and growth. It has also antioxidant properties and plays a role in the healing process [26].

Iron

Multiple biological activities require Fe, which shortage can cause anemia and damage the immune system. Fe deficiency in OSMF could make it severe by reducing the body's capacity to heal damaged tissues and manage inflammation. Fe is the "trace element" that is found in the human body in the highest concentration. The human body contains between three and five grams of Fe, the majority of which is found in blood and the remainder of which is stored as heme in the muscle, bone marrow, and liver [27]. Fe is absorbed in the body and stored in the form of ferritin or hemosiderin. Hemosiderin, a pigment that is produced as a result of the ferritin metabolism, is a dark-orange substance [28].

Biological processes:* *There are numerous Fe-related enzymes, such as *cytochrome a-cp450*, *cytochrome reductases*, *catalase*, *peroxidases*, *xanthine oxidases*, *succinate dehydrogenase*, and *glucose-6-phosphatase*, though the primary Fe-containing compound in the ferrous or ferric state is heme, which is found in *hemoglobin*, *myoglobin*, and other compounds [29].

Cobalt

The main biological function of Co is as a component of vitamin B12, also known as cobalamin, which is necessary for the body's numerous metabolic processes, including the production of red blood cells and the operation of the nervous system. There is some evidence that Co may have indirect impacts on oral health due to its function in vitamin B12 metabolism, although a direct link to OSMF is not well established. Bertrand and Macheboeuf (in 1925) were the first to hypothesize that Co appeared in animal skin. This hypothesis was later supported by other studies that employed spectrographic techniques. A crucial component of the human body, [Co], may be found in both organic and inorganic forms [30]. Between 80 and 300 mcg of vitamin B12 are thought to make up the entire body's Co content.

Biological processes: Cobalamin, another name for vitamin B12, is a water-soluble vitamin. Hydroxycobalamin is transformed by the body into the enzymatically active cofactor forms methylcobalamin and 5'-deoxyadenosylcobalamin [31]. More Co and Fe content in the body lead to the inhibition of thyroid and bone marrow function. These varied *vitamin B12* formulations contain significant levels of Co, a mineral necessary for the synthesis of the amino acids, and other proteins needed for the formation of the myelin sheath.

Chromium

Cr is an essential trace element that has a function in glucose metabolism, namely as part of the glucose tolerance factor, which improves insulin action. While Cr is involved in a variety of metabolic processes in the body, the medical literature does not support a clear link between Cr and OSMF. Cr levels in the body are low. It weighs about 0.006 g in a healthy adult human. Cr hexavalent is a hazardous industrial contaminant. Many lung, GIT, and central nervous system malignancies have been linked to Cr exposure through occupational inhalation. Cr is excreted in the urine and through the skin [32].

Biological processes: Cr regulates blood sugar and cholesterol levels. By enhancing glucose absorption from muscles and other tissues, it leads to an increase in the formation of insulin [33]. The existence of a significant Cr deficit will be sought since Cr is only present in minimal amounts in the human body, and the typical value for serum is between 0.14 and 0.15 ng/mL. Cr and carcinogenesis have been related to lung cancer.

Selenium

Antioxidant trace element Se aids in preventing oxidative cell damage. Oxidative stress contributes to the development of OSMF and influences its course. The body's capacity to prevent oxidative damage may be diminished by Se deficiency, which could make the situation worse

Biological processes: A vital component of selenoproteins, such as glutathione peroxidase, which aids in shielding cells from oxidative damage, is Se. OSMF is linked to oxidative stress, which can harm cells and tissues. Because of its antioxidant properties, Se may be able to lessen the damaging effects of oxidative stress on OSMF-affected oral tissues. The immune system requires Se to operate correctly. It aids in the growth and activation of immune cells, and a lack of it can impede the body's defenses. Immune system dysfunction in OSMF patients may play a role in the onset and development of the illness. Se's potential significance lies in its role in immune function.

Fluorine

Fluoride is well-known for its dental health benefits. It strengthens tooth enamel and helps to prevent tooth decay and cavities. Maintaining proper dental hygiene can be critical in the context of OSMF. Poor dental hygiene and untreated dental disorders, such as cavities and gum disease, can lead to chronic oral irritation and inflammation, thereby exacerbating OSMF. Fluorine is an element of traces that enters the body and is present in minute levels. It is found in drinking water and small amounts in foods.

Biological functions: Fluorine is a chief component of a structured substance found in bones. It also promotes osteoplastic activity when combined with calcium. Fluoride is necessary for tooth decay prevention and tooth enamel strengthening. It stimulates tooth remineralization and aids in cavity prevention. Fluoride can inhibit acid production by bacteria in the mouth, which helps preserve teeth from erosion and decay. Fluoride is a popular element in toothpaste, mouthwash, and other dental treatments to preserve oral health and prevent dental problems.

Water fluoridation: Fluoride is added to drinking water in some areas to give a consistent, community-wide approach to oral health.

OSMF and trace elements

OSMF is a chronic precancerous disorder that is most common in the Southern states. Chewing nuts, eating chili, and having a genetic predisposition have all been suggested as contributing factors. One well-known oral cavity cancer precursor that can result in oral cancer is OSMF. Because oral cancer is so common in developing nations, it is important to identify those who are at risk before the disease becomes widespread, new, or metastatic. The main tools in the battle against oral carcinomas are therefore early detection of these premalignancies and malignant transformation, as well as the role of trace elements in a variety of illnesses. Trace element exposure has been investigated for oral cancers. If they have any bearing on the likelihood of getting oral cancer. The scientific literature on premalignant states is extremely thin. Zn levels in pre-malignancy tissue and serum can be used by researchers to determine the cause of the condition and its best course of treatment.

Signs and symptoms of OSMF

The signs and symptoms of OSMF are restricted mouth opening, burning sensation in the mouth, dryness in the mouth, difficulty in swallowing, progressively decreasing ability to open the mouth, formation of fibrous bands or bands of tissue in the oral cavity, in some cases white patches or leukoplakia on the mucous membrane, change in the color, and texture of the oral mucosa.

Etiology

The etiology of OSMF includes areca nut chewing as a major factor. Other etiological factors include tobacco usage, consumption of chilies and spices, nutritional deficiencies, and genetic factors.

Trace elements disorder in OSMF

It should be mentioned that patients with OSMF do not have different levels of trace elements. It has been found that the rise in OSMF (such as protein and vitamin deficiencies or anemia) is associated with malnutrition, specifically variations in serum Fe, Zn, and Cu levels. Low serum Fe levels can alter the structure of the epithelium, which can worsen barrier function and increase mucosal permeability. Serum ferritin levels have been reported to rise and total iron binding capacity (TIBC) to decrease in OSMF patients based on the histological grade and clinical stage of the disease [34]. According to recent studies, Zn activates superoxide dismutase (SOD), which inhibits the creation of reactive oxygen species (ROS) and prevents fibrosis [35]. Because the oral mucosa is prone to fibrosis and degeneration, a decrease in Zn may hinder the system's ability to remove certain harmful compounds by inhibiting the activity of the peroxidase system. Excessive Cu is present in the tissues of other fibrotic disorders, such as Wilson's disease, pediatric cirrhosis, and primary biliary cirrhosis [36].

One theory links Cu to the formation of OSMF by suggesting that Cu increases the production of collagen and acts as a cofactor of lysyl oxidase. It has been shown that adding Cu to fibroblasts in vitro boosts their ability to proliferate [35]. A study using human skin fibroblasts demonstrated the cells' effective absorption of Cu. Intracellular Cu may form tissue complexes via the lysyl oxidase pathway, which subsequently promotes collagen synthesis and cross-linking [36]. A positive correlation between the incidence of OSMF and the Cu content of drinking water was discovered through extensive case-control research. When exposed to a diet that contains slightly more Cu than the average diet, like drinking Cu water, the concentration of Cu in local tissues increases, increasing the risk of OSMF formation in the oral mucosa. Furthermore, it has been suggested that the development of OSMF syndrome impairs Cu elimination [35]. Furthermore, in the blood and saliva of patients with OSMF, immunoglobulins G and A levels increased while hemoglobin (Hb) and total serum protein (TSP) levels sharply decreased.

Oral precancerous and cancer trace elements

In India, oral cancer is much more prevalent than it is worldwide. Leukoplakia is the most common precancerous condition, amounting roughly to 85%. Oral cancer can result from a multitude of factors, such as alcohol consumption, infectious agents, genetic modifications, fungal infections, and persistent inflammation. Trace elements are multifunctional anti-cancer medications that support various biological processes. Lower levels of Zn and Cu were discovered. More researchers are noticing a possible connection between trace metal levels in a patient's blood and cancer mortality when the patient has been diagnosed with head and neck cancer. Regarding the beginning and development of carcinogenesis, the [Cu]-to-[Zn] ratio is also considered a legitimate prodrug. The trace elements Zn and Cu are part of the human body's anti-carcinogenic defense system. In several investigations, Cu is a component of many enzymes involved in cell metabolism, especially those that are involved in the oxidation process. Patients with oral premalignant and malignant lesions and disorders had higher mean serum Cu levels. Previous studies found that the groups with oral leukoplakia and oral squamous cell carcinoma (OSCC) had higher blood concentrations of Cu. According to a recent study, blood [Cu] levels in patients with OSMF increased steadily as they progressed through the clinical stages.

The Cu-Zn SOD enzyme, a part of every vertebrate's primary antioxidant system, needs Zn as a cofactor. Research has indicated that serum Zn levels were lower in people with potentially premalignant diseases, such as oral leukoplakia. This could be due to Zn consumption in response to oxidants produced by tobacco use or the high Cu content of areca quid metabolism [37]. Researchers believe that Zn limits the ability of malignant prostate cells to migrate and invade, even though there was no appreciable difference in serum Zn levels between the oral leukoplakia and OSMF classes. Fibrosis results from an increase in choline penetration brought on by decreased epithelial vascularity. Because Fe is involved in the production of collagen, reduced Fe content has been associated with Fe shortage in patients with OSMF. Fe deficiency is thought to be caused by a burning sensation and food shortage brought on by erosions in patients with OSMF, as well as an increase in tumor load in patients with OSCC.

Further studies on the concentrations of these micronutrients in precancerous and malignant tissue, along with their relationship to changes in serum, will help identify their involvement in the development of oral carcinogenesis. As oral cancer has progressed, they have proven to be effective biological markers.

Discussion

A persistent, evasive, and debilitating condition of the oral mucosa, OSMF is characterized by epithelial atrophy and a buildup of collagen fibers in the lamina propria and submucosa. OSMF, an illness of the oral cavity that may be malignant, is highly prevalent in Asian nations. It is critical to diagnose and treat OSMF based on its stage because it is believed to be a precancerous illness [38]. Serum Cu levels were found to be higher than serum Fe levels, according to studies. Furthermore, in line with the study's findings, a clear pattern was noticed, with the serum Cu level rising steadily as the histological grade and clinical stage of OSMF progressed. As a result, serum Cu levels and the degree of fibrosis may be directly correlated.

An essential cofactor for the expression of lysyl oxidase is Cu. The high levels of Cu in areca nuts, a major cause of OSMF, stimulate fibrogenesis by upregulating lysyl oxidase and obstructing the breakdown of collagen. The rise in serum Cu levels could be explained by either a decrease in the catabolism of serum ceruloplasmin or an increase in the liver's production of Cu-containing ceruloplasmin as a result of an inflammatory response [9]. It has also been proposed that superoxide radicals or other reducing agents, such as ascorbate, which weaken the copper complex, may mediate the mechanism through which cellular damage caused by Cu ions occurs. The Fe used during the fibrosis process may be the cause of the decreasing Fe content [39]. The prevalence of Fe-deficient anemia may have been influenced by the clinical features of OSMF. Eating solid food is unpleasant due to the initial burning sensation, vesiculation, and ulceration. This leads to insufficient intake of a healthy diet, which may ultimately cause anemia. Reduced vascularity is also a consequence of tissue Fe deficiency [40]. Cytochrome oxidase is a Fe-containing enzyme that is required for normal epithelium maturation. In Fe deficiency anemia, low levels of this enzyme result in epithelium atrophy and lack of maturation.

## Conclusions

The body requires optimal availability of these elements for proper physiological functioning, even though they are only found in trace amounts. They are essential to the biodynamic maintenance of the body. Both excess and deficiency conditions initiate, develop, and stimulate many disease processes. The trace elements have been covered in great detail in this study. OSMF is a sophisticated precancerous syndrome affecting the oral cavity and the oropharynx. Once fibrosis has developed, there is no cure, and there is a significant risk that the condition may progress to cancer. Aggressive steps should be taken for an early diagnosis to improve the prognosis. A biopsy is a gold standard for diagnosing OSMF, but it is an invasive and time-consuming procedure. Many recent developments are now being used to identify OSMF in a very early stage and prevent it from developing into a late and irreversible stage. The results of this study support the idea that trace elements could be utilized for diagnosing and predicting OSMF in patients. However, more research on a more diverse population is anticipated to bolster the theory. These endeavors may prove beneficial in the early identification and management of high-risk demographics, in addition to detecting additional premalignant lesions possessing malignant potential.
